# Oral Health-Related Quality of Life in Italian Children and Adolescents Living with Bone Dysplasia: A Cross-Sectional Study

**DOI:** 10.3390/ijerph21030306

**Published:** 2024-03-06

**Authors:** Patrizia Defabianis, Rossella Ninivaggi, Daniele Tessaris, Norma Bocca, Federica Romano

**Affiliations:** 1 Department of Surgical Sciences, C.I.R. Dental School, University of Turin, 10126 Turin, Italy; rossella.ninivaggi@unito.it (R.N.); norma.bocca@unito.it (N.B.); federica.romano@unito.it (F.R.); 2 Department of Pediatric Endocrinology, Regina Margherita Children’s Hospital-Health and Science, 10126 Turin, Italy; daniele.tessaris@unito.it; 3 Department of Public Health and Pediatrics, University of Turin, 10126 Turin, Italy

**Keywords:** oral health-related quality of life, bone dysplasia, rare diseases, OHIP-14, COHIP, SF-CPQ, children

## Abstract

Bone dysplasia (BD) refers to a group of rare disorders characterized by skeletal and dental anomalies which may negatively influence oral health-related quality of life (OHRQoL). The aim of this cross-sectional study was to assess the impact of BD on OHRQoL in Italian children and adolescents and to assess whether gender and age influence their OHRQoL. A total of 40 patients with BD and 40 age- and gender-matched controls (aged 8–14 years) were asked to complete the Oral Health Impact Profile-14 (OHIP-14), Child Oral Health Impact Profile (COHIP), and the short form of the Child Perceptions Questionnaire (SF-CPQ). Children with BD showed statistically significant lower overall scores of all the questionnaires than the controls (all *p* < 0.001), with the largest differences being detected in overall symptoms, functional well-being, and social well-being domains. While no statistically significant gender-related differences were observed, adolescents aged 11–14 years experienced worse perception in the emotional and social well-being SSF-CPQ domains (*p* = 0.042 and *p* = 0.045, respectively) and in the peer interaction COHIP domain (*p* = 0.011) compared to the younger age group. Based on these findings, children suffering from BD experience poorer OHRQoL than their healthy peers, suggesting that oral and dental issues may be of special importance for the socio-psychological well-being of these growing individuals.

## 1. Introduction

Bone dysplasia (BD) comprises a group of rare genetic diseases (affecting less than 1 in 200,000) characterized by abnormalities in bone development and structure [1]. These disorders often appear during childhood and can result not only in various skeletal deformities but also in oral manifestations, potentially affecting the individual’s well-being [2]. The most common forms are X-linked hypophosphatemic rickets (XLH), osteogenesis imperfecta (OI), McCune–Albright syndrome (MAS), fibrous dysplasia (FD), and Pseudohypoparathyroidism (PHP) [3]. XLH is characterized by disruptions in phosphate homeostasis, leading to the defective mineralization of bone and teeth [4]. In contrast, OI is a collagenopathy identified by structural abnormalities in osseous and dental tissues and may involve varying degrees of dentinal dysplasia [5,6]. In addition to skeletal dysplasia, FD is characterized by increased risk of dental fractures, and MAS may also exhibit facial asymmetry, dental malocclusion, as well as dental anomalies (dentine dysplasia, enamel hypoplasia), with high vulnerability to cavities [7,8]. Finally, PHP may present enamel aplasia and/or hypoplasia, incomplete dentin mineralization, short and blunt roots, delayed eruptions, and missing or impacted teeth [3].

Each of these disorders represents unique challenges in terms of diagnosis, management, and treatment, highlighting the complexity of bone development and the importance of understanding these conditions for effective medical care [4,5,6]. In 2009, the Council of the European Union issued a recommendation to member states to develop measures to improve medical care for people with rare diseases [9,10].

On average, it takes seven years before a rare disease is correctly diagnosed [11]. In the process, its oral manifestations seem to negatively influence oral health-related quality of life (OHRQoL) [12]. With oral symptoms, the dentist can contribute towards reducing the time taken for a diagnosis. Dental checkups make up one of the most frequent reasons for contact between patients and healthcare professionals. In cases of oral symptoms, the dentist is often the first healthcare professional who is able to identify a rare disease by its oral manifestations. This could in turn positively influence OHRQoL and also overall quality of life because dentists and physicians can start the appropriate therapy earlier [13,14,15]. Thus far, there have been very few studies addressing OHRQoL concerns among individuals with BDs [6], most of them focused on the adult population, and none have been conducted in Italy. There are differences between how children and adults experience the impact of oral health on their quality of life, emphasizing the need to assess this aspect in younger age groups. OHRQoL perceptions have been found to also differ according to gender and disease severity [16,17].

Therefore, the aim of the present observational study was to assess OHRQoL in Italian children and adolescents suffering from BDs compared to their healthy peers. As a secondary outcome, this study explored if children with BDs from different age groups and genders perceive OHRQoL differently. The overall goal is to provide information for parents, clinicians, and public health professionals in order to better tailor the management of children living with BD towards their specific need.

## 2. Materials and Methods

This cross-sectional study was carried out at the Section of Pediatric Dentistry at the Dental School of the University of Turin (Italy) from June to November 2023, and it was reported to be in accordance with the guidelines of the STROBE Statement (Table S1). Children or adolescents of both genders, from the age of 8 to 14 years, with a confirmed genetic diagnosis of BD, were invited to participate in the study. They received care at the Regina Margherita Hospital, a highly specialized center for rare diseases in North Italy, where they were followed-up by the same pediatric endocrinologist. Once participants with BD were enlisted, age-balanced, and gender-matched, healthy children were identified from the hospital database and consecutively recruited for the control group while attending routine scheduled appointments. Only Italian-speaking participants or foreigners able to read the Italian language fluently were enrolled. Patients with concurrent systemic pathologies or uncapable of understanding the questionnaires were excluded from the study. Recruitment was scheduled to run for approximately 6 months, aiming to enroll at least 35 participants per group.

The study was conducted in accordance with the Declaration of Helsinki and approved by the Institutional Ethics Committee of “AOU Città della Salute e della Scienza”, Turin (protocol number 0068999; date of approval: 5 June 2023). Informed written consent was obtained from each participant’s parent or legal guardian before enrollment.

### 2.1. Data Collection

Parents of both groups answered a questionnaire about demographics, including participant’s age, gender, race, and parent’s educational attainment. In addition, each parent/caregiver of children suffering from BD filled out a standardized form about genetic diagnosis, pathological familiarity, type of birth, perinatal pathologic findings, child bone fracture experience, pain, surgery, and pharmacological therapy.

OHRQoL was evaluated using the Italian-validated version of the Oral Health Impact Profile-14 (OHIP-14), Child Oral Health Impact Profile (COHIP), and the short form of the Child Perceptions Questionnaire (SF-CPQ). Participants of both groups were asked to complete the questionnaires without any parental support. Each questionnaire was then checked in order to avoid missing data.

The OHIP-14 questionnaire [18] includes 14 items, divided into seven domains, about the impact of oral health problems on the individual’s life during the past month. The seven domains include functional limitation, physical pain, psychological discomfort, physical disability, psychological disability, social disability, and social handicap [15]. All items are evaluated on a Likert scale at 5 points from 0 to 4 (never = 0; almost never = 1; occasionally = 2; quite often = 3; and very often = 4). The score of each dimension, consisting of two questions, may vary from 0 to 8. The overall OHIP-14 score is comprised from 0 to 56, with higher values reflecting lower OHRQoL.

The COHIP questionnaire is organized into five subscales (oral symptoms, functional well-being, social/emotional well-being, school environment, and peer interaction) for 34 items referring to children’s positive or negative experiences in the last 3 months. Every item is rated on a five-point Likert scale (from 0 = never to 4 = almost all the time) with the additional response option ‘I do not know’, that is scored as missing. The subscale scores are added together to give the overall OHRQoL, ranging from 0 (the worst OHRQoL) to 136 (the best OHRQoL) [19].

The SF-CPQ comprises 8 items and represents 4 conceptual domains: oral symptoms (OS1: ‘Pain’ and OS2: ‘Food stuck or caught’), functional limitation (FL1: ‘Difficulty biting or chewing firm food’ and FL2: ‘Taken longer than others to eat a meal‘), emotional well-being (EW1: ‘Been upset because of problems with your teeth, lips, mouth or jaws’ and EW2: ‘Been irritable or frustrated because of problems with your teeth, lips, mouth or jaws’), and social well-being (SW1: ‘Missed school because of problems with your teeth, lips, mouth or jaws’ and SW2: ‘Not wanted to talk to other children because of problems with your teeth, lips, mouth or jaws’). Each item is ranked on a 5-point Likert scale ranging from 1 to 5 (1 = never, 2 = once or twice, 3 = sometimes, 4 = often, and 5 = very often). Summary scores ranged from 0 to 16, with higher total scores indicating worse OHRQoL [20].

### 2.2. Statistical Analysis

All data were anonymized, and each patient was identified by a code. Data were analyzed using statistical software (SPSS, version 28, IBM Corp., Armonk, NY, USA) and published as aggregates. Total and sub-scale scores of all the questionnaires were compared with those of the questionnaires filled by the control group; questionnaires where less than 75% of it was completed were excluded from the evaluation. The internal consistency reliability of the questionnaires was confirmed by Cronbach’s alpha coefficient.

For quantitative data, descriptive statistics reported mean and standard deviation (SD), as well as median and range. Qualitative data were summarized using absolute and relative frequencies. Bivariate statistical analysis included the Chi-square test or Fisher’s exact test for categorical variables and Student’s *t*-test for quantitative variables. The Mann–Whitney U-test test was used for the comparison of continuous asymmetric distributions. Finally, prespecified subgroup analyses were conducted to detect any difference in OHRQoL among patients with BD according to age (categorized as 8–10 years and 11–14 years), gender, and type of BD. Due to the exploratory nature of these secondary analyses, multiplicity was not corrected. The null hypothesis was that there is no difference in OHRQoL scores in the comparison between the BD and control groups. Furthermore, it was hypothesized that neither gender, age, nor type of BD impacted the OHRQoL domain scores. A *p*-value < 0.05 was considered statistically significant.

## 3. Results

### 3.1. Demographic and Medical Features of the Sample

Overall, 80 participants were included: 40 BD patients and 40 controls. The flow chart of the study is reported in Figure S1. The demo-graphic and genetic data are shown in Table 1. The gender ratio (male/female) was 21/19 in both groups; the mean age was 11.1 years (±2.5) in the BD group and 10.8 years (±2.2) in the control group; and the difference in mean age was not statistically significant (*p* = 0.538). All participants were white, except for two children in the BD group. No differences were observed in the family educational level between the two groups (*p* = 0.581).

In the BD group, the most prevalent disorder was OI (60.0%), followed by PHP (15.0%) and XHL (12.0%). Familiarity was found in 24 out of 40 patients (60.0%), with the majority of them (62.5%) having inherited BD through the maternal line. Only one fifth of the patients (22.5%) were delivered via caesarean. Dentinogenesis imperfecta (DI) was present in four individuals (10.0%). Half of the patients had experienced at least one fracture in their life, and eight (20.0%) had at least one surgery. Eight of the patients with OI reported bisphosphonate intake (two patients took pamhydrinate and six neridronate).

Regarding pain perception, as summarized in Table 2, 32.5% of patients with BD experienced localized or diffuse bone pain, with most of them suffering from OI.

### 3.2. OHRQoL Questionnaires

A total of 80 SF-CPQ questionnaires, 80 COHIP questionnaires, and 80 OHIP-14 questionnaires were completed by the participants. The internal consistencies of the questionnaires were good; Cronbach’s alpha of OHIP-14 was 0.820 for BD patients and 0.848 for the controls, and similar values were obtained for SF-CPQ (0.823 vs. 0.827) and COHIP (0.847 vs. 0.813).

The comparison analysis of all the questionnaire scores between the two groups is shown in Table 3. Overall, the mean total score of OHIP-14 was significantly higher in the BD group (5.82 ± 6.69) in comparison to the mean score of the controls (2.4 ± 3.9) (*p* = 0.011), meaning that patients affected by BD had a poorer OHRQoL. Specifically, the items of the OHIP-14 with mean scores which were significantly higher in the BD group were “physical pain”, “psychological discomfort”, “psychosocial disability”, and “social disability” (*p* = 0.025, 0.003, 0.026, and 0.015) compared to those of the control group.

When considering the SF-CPQ questionnaire, the BD group presented statistically significant higher scores in all the SF-CPQ items compared to the controls, namely in the “oral symptoms”, “functional well-being”, and “emotional well-being” items, resulting in less favorable OHRQoL (all *p*-values < 0.001). Indeed, the overall lower value for the COHIP questionnaire showed poorer OHRQoL for BD patients with respect to the controls, with the largest differences being detected in “overall symptoms”, “functional well-being”, and “social/emotional well-being” domain-specific scores (all *p*-values < 0.001).

As secondary analysis, the impact of gender and age on OHRQoL perception among BD children was explored, and data are summarized in Table 4, Table 5 and Table 6. Although females reported higher values in the overall scores and in almost all the items of both the OHIP-14 (Table 4) and SF-CPQ (Table 5) questionnaires, these differences were not statistically significant compared to males (*p* > 0.05).

With regard to the COHIP questionnaire (Table 6), its scores tended to overlap in the female and male groups.

Significant differences were identified based on the child’s age in both the SSF-CPQ and COHIP questionnaires (Table 5 and Table 6), but not in the OHIP-14 questionnaire (Table 4). Older children experienced worse perception in the “emotional well-being” and “social well-being” SSF-CPQ domains compared to the younger age group (*p* = 0.042 and *p* = 0.045, respectively), as well as in the “peer interaction” COHIP domain (*p* = 0.011). For the other domains, score differences were not statistically significant (*p* > 0.05).

Finally, the impact of type of BD on OHRQoL was assessed. As the number of participants with MAS and FD was too small to conduct a meaningful statistical analysis, only descriptive data are reported in Table 7. The overall scores of the three questionnaires consistently suggested poorer OHRQoL among children with PHP.

## 4. Discussion

This is the first study analyzing OHRQoL among Italian children and adolescents suffering from BD compared to healthy peers. Poorer OHRQoL was observed, irrespective of the questionnaire used, with greater impairment of the psychosocial and functional domains. Most studies available in the literature focused on the adult population or compared the child’s OHRQoL across clinical subtypes of the same BD [6,21]. In this study, each participant had a control, matched for age and gender, who were also recruited from the same hospital, allowing participants to be compared with patients of a similar background.

Although BDs have different etiology and pathophysiology, the common denominator across all these conditions is their tendency to manifest early in life, significantly impacting dental and jaw structures. This, in turn, negatively affects oral functional abilities, mastication, aesthetics, and social life during childhood and adolescence [6], thereby influencing the OHRQoL for these patients. The relation between oral health status and quality of life is a complex issue, especially in these age groups. Adult people assess their OHRQoL by comparing their expectations and experiences [22], but children do not account for their expectations of oral health when they try to score the negative impact of the disease based on their perception. Moreover, young patients may be at different phases of the disease when they are filling out the questionnaire [22,23]. Considering that there is still no consensus on which set of questionnaires may be more appropriate for BD, children received three questionnaires, each of which highlighted specific aspects through questions underlying different domains. Thus, the short form of the OHIP and CPQ questionnaires were chosen to ensure that they were not overwhelmed by too many questions [17], while the full form of the COHIP was more complete and it included more specific questions. Overall, the SF-CPQ, COHIP, and OHIP-14 questionnaires explore the domains that contribute to evaluating the child’s quality of life through questions that investigate the main oral aspects.

In our study, the total score of the three questionnaires was consistently worse in the dysplasia group; even if this was due to the small number of participants with MAS and FD, a comparison among the different types of BD was not allowed. These findings can only be partly supported by what is referred to in the literature. Gjørup et al. [16] observed poorer OHRQoL in OI and XLH adult patients using the OHIP-49 questionnaire compared to adults in the Danish population who were not patients of these diseases. Adults with XLH scored worse than adults with OI in the domains of functional limitation, psychological discomfort, and physical disability, probably because of the high prevalence of endodontically affected painful teeth, aesthetically compromising DI and mandibular overjet. Cachia Mintoff et al. [17] reported a worsening of the COHIP-SF scores as OI severity increases in children aged 8–16 years in the U.K. This finding was confirmed by Najirad et al. [22] in American adolescents aged 11 to 14 years, but not in children aged 8 to 10 years, using the CPQ questionnaire. The authors attributed the significantly higher grade of functional limitation in OI types III and IV to the highest occurrence of class III malocclusion and DI among these two OI subtypes.

In the present study, in addition to functional limitations and psychosocial impairment, pain would seem to have a quantitatively higher negative impact on child’s OHRQoL across all three questionnaires. Questions relating to pain are present in the pain domain of the OHIP-14 (Have you had any annoying pain in your mouth?) as well as in the oral health domain of the COHIP (Do you suffer from toothaches?) and in the oral symptom domain of the SF-CPQ (In the last 3 months, how often have you had pain in your teeth, lips, mouth or jaw?). It may be hypothesized that pain is secondary to the dental pathological aspects which can be observed in different clinical phenotypes of fibrous dysplasia.

Regarding self-esteem and social well-being, we can hypothesize, as Mintoff did, that children with BD have a social network influenced by various factors, such as the inability to engage in risk-prone activities for fear of fractures while playing with their peers and the need for frequent hospital visits [17]. Specifically, feeling different from their peers may lead the child to have a distinct perception of their OHRQoL. Interestingly, the school domain in the COHIP questionnaire would seem not to be affected, likely because school-related issues are common among children regardless of pathology.

As far as gender is concerned, in the present study, males and females were equally affected by BD, and this result is confirmed by other studies reported in the literature [24]. Females had worse scores than males in the SF-CPQ and OHIP-14 questionnaires, in contrast with the results reported by Cachia Mintoff et al. [17]; regardless, the difference found was not statistically significant. Furthermore, according to Cachia Mintoff et al., minority groups may affect the overall results of the questionnaires, as people from different ethnicities may have different ideas or priorities for their quality of life. In our study group, only two Black American patients were included, but with no remarkable impact on the general results found [17].

With regard to age differences in OHRQoL perception, age seems to impact not only in terms of psychological but also relational and self-esteem aspects. Indeed, the emotional and social well-being domains of the SF-CPQ and the self-confidence domain of the COHIP scored worse in adolescents than in patients under 10 years of age. In line with these findings, in Najirad’s study [6], age had a bigger impact on OHRQoL in adolescents than patients under 10 years of age, probably because they are more aware of the functional problems that they have had to live with for longer. This is in disagreement with the study by Ierardo et al. [24], which concluded that adolescents can adapt psychologically to the circumstances related to their pathology, and that in adolescents with BD, OHRQoL is the result more of physical and functional characteristics rather than emotional and psychological problems. Nonetheless, it is reasonable to assume that the presence of aesthetically compromised incisors affected by DI can seriously influence the OHRQoL of children and adolescents, resulting in social and psychological disability [16].

The main limit of this study is the sample size, even if is relatively large compared to previous studies focusing on questionnaires. Due to the rarity of BD, patients with different clinical characteristics and therapies were grouped together in order to ensure that a reasonable number of participants were recruited to the study. As we evaluated the OHRQoL of children with BD using the SF-CPQ questionnaire with was developed for children from 8 to 14 years, we restricted enrollment only to children and adolescents within this age interval. This may limit the generalizability of the present findings considering that life experiences and perspectives can vary greatly based on age. Furthermore, the questionnaires administered, even if largely applied, have not been previously validated for patients diagnosed with rare disorders.

In addition, no multiplicity adjustment was applied to secondary outcome analyses; therefore, because of the potential of type I errors, the findings should be regarded as exploratory. The impact of the drugs taken by some patients was not examined due to the small sample of patients taking bisphosphonates present in the study. Bisphosphonates decrease fracture incidence, which results in decreased surgical interventions and hospitalization [25], but according to Oliveira et al., alendronate probably interferes in the secretory pathways of parotid and submandibular glands [26], with an important effect on the oral health and consequently on OHRQoL [27]. Finally, because of its cross-sectional design, the study was not able to capture age-related differences in OHRQoL perceptions over time.

## 5. Conclusions

Based on the consistent results acquired across the questionnaires, children with BD experience poorer OHRQoL than their healthy peers. This suggests that oral and dental issues may be of special relevance for the sociopsychological well-being of these growing individuals. The perception of oral health may vary by age, but not by gender, for children and adolescents suffering from BD. However, due to the exploratory nature of such findings, larger-scale multi-center studies are needed to make definitive conclusions. Future research could also contribute to a deeper understanding of the influence of specific oral manifestations on the OHRQoL of these young patients and to the identification of promising directions for tailored interventions in this little-studied cohort.

## Figures and Tables

**Table 1:** Demographic data and genetic diagnosis by group.

Variables	Bone Dysplasia (*n* = 40)	Controls (*n* = 40)	*p*-Value
Age (years), mean ± SD	11.1 ± 2.5	10.8 ± 2.2	0.538
Gender, *n* (%)			NA
Female	19 (47.5)	19 (47.5)	
Male	21 (52.5)	21 (52.5)	
Race, *n* (%)			0.494
White	38 (95.0)	40 (100)	
Black or other	2 (5.0)	0 (0.0)	
Parents’ education *n*, (%)			0.581
High	8 (20.0)	12 (30.0)	
Medium	20 (50.0)	18 (45.0)	
Low	12 (30.0)	10 (25.0)	
BD diagnosis, *n* (%)			
OI	24 (60.0)	-	
XHL	5 (12.5)	-	
MAS	2 (5.0)	-	
FD	3 (7.5)	-	
PHP	6 (15.0)	-	
Familiarity for BD, *n* (%)			
Mother line	15 (37.5)	-	
Father line	5 (12.5)	-	
Both parents	4 (10.0)	-	
No familiarity	16 (40.0)	-	

BD, bone disorder; OI, osteogenesis imperfecta OI; XHL, X-linked hypophosphatemic rickets; MAS, McCune–Albright syndrome; FD, fibrous dysplasia; PHP, pseudohypoparathyroidism, SD, standard deviation; NA, not applicable.

**Table 2:** Bone pain according to the genetic diagnosis of bone dysplasia (*n*, %).

	OI (*n* = 24)	XHL (*n* = 5)	PHP (*n* = 6)	MAS (*n* = 2)	FD (*n* = 3)	Total (*n* = 40)
No pain	15 (62.5)	4 (80.0)	3 (50.0)	2 (100)	3 (100)	27 (67.5)
Localized pain	5 (20.8)	1 (20.0)	2 (33.33)	0 (0.0)	0 (0.0)	8 (20.0)
Diffuse pain	3 (12.5)	0 (0.0)	1 (16.7)	0 (0.0)	0 (0.0)	4 (10.0)
Occasional pain	1 (4.2)	0 (0.0)	0 (0.0)	0 (0.0)	0 (0.0)	1 (2.5)

**Table 3:** Overall and subscale OHIP-14, SF-CPQ, and COHIP scores by group.

Questionnaires	Group				*p* Value
OHIP-14 domains (maximum possible score)	Bone dysplasia patients (*n* = 40)		Controls (*n* = 40)		
	Mean ± SD	Median (min/max)	Mean ± SD	Median (min/max)	
Overall OHIP (56)	5.82 ± 6.69	3.0 (0/22)	2.40 ± 3.90	0.0 (0/21)	0.011
Functional limitation (8)	0.45 ± 1.19	0.0 (0/6)	0.50 ± 0.93	0.0 (0/4)	0.272
Physical pain (8)	1.85 ± 1.77	2.0 (0/6)	1.05 ± 1.58	0.0 (0/5)	0.025
Phycological discomfort (8)	1.12 ± 1.92	0.0 (0/8)	0.17 ± 0.54	0.0 (0/2)	0.003
Physical disability (8)	0.62 ± 1.54	0.0 (0/7)	0.32 ± 0.97	0.0 (0/5)	0.504
Psychosocial disability (8)	1.10 ± 1.67	0.0 (0/5)	0.25 ± 0.54	0.0 (0/2)	0.026
Social disability (8)	0.40 ± 0.95	0.0 (0/4)	0.075 ± 0.47	0.0 (0/3)	0.015
Social handicap (8)	0.27 ± 0.78	0.0 (0/3)	0.02 ± 0.15	0.0 (0/1)	0.084
SF-CPQ domains (maximum possible score)	Bone dysplasia patients (*n* = 40)		Controls (*n* = 40)		
	Mean ± SD	Median (min/max)	Mean ± SD	Median (min/max)	
Overall SF-CPQ (16)	7.40 ± 6.40	6.0 (0/32)	1.50 ± 2.80	0.0 (0/12)	<0.001
Oral symptoms (4)	2.85 ± 1.91	3.0 (0/8)	0.62 ± 1.07	0.0 (0/4)	<0.001
Functional well-being (4)	1.77 ± 2.24	1.0 (0/8)	0.40 ± 1.13	0.0 (0/4)	<0.001
Emotional well-being (4)	1.52 ± 1.85	1.0 (0/8)	0.15 ± 0.48	0.0 (0/2)	<0.001
Social well-being (4)	1.27 ± 1.83	0.0 (0/8)	0.22 ± 0.69	0.0 (0/3)	0.002
COHIP domains * (maximum possible score)	Bone dysplasia patients (*n* = 40)		Controls (*n* = 40)		
	Mean ± SD	Median (min/max)	Mean ± SD	Median (min/max)	
Overall COHIP (136)	108.37 ± 12.80	110.5 (82/135)	126.25 ± 8.08	128.0 (106/136)	<0.001
Oral symptoms (40)	30.17 ± 5.95	30.5 (15/39)	36.6 ± 3.84	37.0 (26/40)	<0.001
Functional well-being (24)	18.60 ± 3.56	19.0 (9/24)	22.65 ± 1.95	23.0 (16/24)	<0.001
Emotional well-being (32)	27.50 ± 4.41	28.5 (13/32)	31.82 ± 0.67	32.0 (28/32)	<0.001
School environment (16)	14.92 ± 1.43	16.0 (12/16)	15.32 ± 1.20	16.0 (12/16)	0.159
Peer interaction (24)	17.17 ± 5.12	17.5 (6/24)	19.85 ± 3.41	20.0 (14/24)	0.020

Oral Health Impact Profile-14 (OHIP-14), Child Oral Health Impact Profile (COHIP), short form of Child Perceptions Questionnaire (SF-CPQ); * higher COHIP values denote better OHRQoL.

**Table 4:** Overall and subscale scores of Oral Health Impact Profile-14 in bone dysplasia patients by gender and age.

Group	OHIP-14 Score [Mean ± SD, Median, Range)]							
	Overall	Functional Limitation	Physical Pain	Phycological Discomfort	Physical Disability	Psychosocial Disability	Social Disability	Social Handicap
Gender								
Female (*n* = 19)	6.9 ± 7.9 4.0 (0/22)	0.21 ± 0.71 0.0 (0/3)	1.78 ± 1.98 1.0 (0/6)	1.42 ± 1.98 0.0 (0/6)	1.05 ± 1.98 0.0 (0/7)	1.42 ± 1.80 0.0 (0/5)	0.57 ± 0.16 0.0 (0/4)	0.47 ± 1.02 0.0 (0/3)
Male (*n* = 21)	4.8 ± 5.3 3.0 (0/19)	0.60 ± 1.49 0.0 (0/6)	1.90 ± 1.60 2.0 (0/6)	0.85 ± 1.87 0.0 (0/8)	0.23 ± 0.88 0.0 (0/4)	0.8 ± 1.47 0.0 (0/4)	0.23 ± 0.7 0.0 (0/3)	0.89 ± 0.43 0.0 (0/2)
*p*-Value	0.830	0.469	0.688	0.307	0.226	0.282	0.486	0.376
Age								
8–10 yrs (*n* = 18)	5.8 ± 6.5 3.5 (0/20)	0.22 ± 0.64 0.0 (0/2)	2.16 ± 1.82 2.0 (0/6)	0.83 ± 1.54 0.0 (0/6)	1.16 ± 2.09 0.0 (0/7)	0.83 ± 1.42 0.0 (0/5)	0.44 ± 1.04 0.0 (0/4)	0.22 ± 0.73 0.0 (0/3)
11–14 yrs (*n* = 22)	5.7 ± 6.96 2.5 (0/22)	0.63 ± 1.49 0.0 (0/6)	1.59 ± 1.73 1.5 (0/6)	1.36 ± 2.19 0.0 (0/8)	0.18 ± 0.66 0.0 (0/3)	1.31 ± 1.86 0.0 (0/5)	0.36 ± 0.99 0.0 (0/3)	0.31 ± 0.83 0.0 (0/3)
*p*-Value	0.717	0.527	0.325	0.563	0.172	0.563	0.840	0.882

**Table 5:** Overall and subscale scores of short form of Child Perceptions Questionnaire in bone dysplasia patients by gender and age.

Group	SF-CPQ Scores [Mean ± SD, Median, (Range)]				
	Overall SF-CPQ	SF-CPQ Oral Symptoms	SF-CPQ Functional	SF-CPQ Emotional Well-Being	SF-CPQ Social Well-Being
Gender					
Female (*n* = 19)	9.2 ± 8.0 7.0 (0/32)	3.47 ± 2.11 3.0 (0/8)	2.36 ± 2.81 1.0 (0/8)	1.78 ± 2.07 1.0 (0/8)	1.57 ± 2.26 1.0 (0/8)
Male (*n* = 21)	5.8 ± 4.13 5.0 (0/14)	2.28 ± 1.55 2.0 (0/5)	1.23 ± 1.44 1.0 (0/5)	1.28 ± 1.64 0.0 (0/4)	1.00 ± 1.34 0.0 (0/4)
*p*-Value	0.226	0.083	0.307	0.405	0.573
Age					
8–10 yrs (*n* = 18)	5.88 ± 5.1 4.5 (0/18)	2.72 ± 1.90 3.0 (0/8)	1.66 ± 2.30 0.5 (0/8)	0.83 ± 1.24 0.0 (0/4)	0.66 ± 1.23 0.0 (0/4)
11–14 yrs (*n* = 22)	8.68 ± 7.20 8.0 (0/32)	2.95 ± 1.96 3.0 (0/8)	1.86 ± 2.25 2.0 (0/8)	2.09 ± 2.09 2.0 (0/8)	1.77 ± 2.11 2.0 (0/8)
*p*-Value	0.163	0.717	0.563	0.042	0.045

**Table 6:** Overall and subscale scores of Child Oral Health Impact Profile in bone dysplasia patients by gender and age.

Group		COHIP Scores [Mean ± SD, Median, (Range)] *				
	Overall COHIP	Oral Symptoms	Functional Well-Being	Social- Emotional Well-Being	School Environment	Peer Interaction
Gender						
Female (*n* = 19)	106.21± 14.68 110.0 (82/135)	30.00 ± 6.61 31.0 (15/39)	17.89 ± 4.70 18.0 (9/24)	26.63 ± 4.58 28.0 (13/32)	14.94 ± 1.54 16.0 (12/16)	16.73 ± 5.98 17.0 (6/24)
Male (*n* = 21)	110.33 ± 10.82 111.0 (88/129)	30.3 ± 5.45 30.0 (18/38)	19.24 ± 3.18 20.0 (11/24)	28.29 ± 4.09 29.0 (19/32)	14.90 ± 1.37 16.0 (12/16)	17.57 ± 4.31 18.0 (9/24)
*p*-Value	0.376	1.000	0.145	0.169	0.810	0.768
Age						
8–10 yrs (*n* = 18)	111.44 ± 11.90 113.5 (82/129)	30.05 ± 6.23 30.5 (15/38)	18.72 ± 3.22 19.5 (9/23)	28.11 ± 3.22 28.5 (21/32)	15.11 ± 1.40 16.0 (12/16)	19.44 ± 4.21 20.0 (12/24)
11–14 yrs (*n* = 22)	105.86 ± 13.22 109.0 (82/135)	30.27 ± 5.86 30.5 (18/39)	18.5 ± 3.81 19.0 (11/24)	27.0 ± 5.16 28.5 (13/32)	14.77 ± 1.47 15.5 (12/16)	15.31 ± 5.13 15.0 (6/24)
*p*-Value	0.100	1.000	0.798	0.840	0.476	0.011

* higher COHIP values denote better OHRQoL.

**Table 7:** Overall scores of the questionnaires by type of bone dysplasia [mean ± SD, median, (range)].

BONE DYSPLASIA	OHIP-14	SF-CPQ	COHIP *
OI (*n* = 24)	5.87 ± 5.75 4.0 (0/20)	6.83 ± 7.09 5.5 (0/32)	110.21 ± 13.28 112.50 (82/135)
XLH (*n* = 5)	4.80 ± 6.42 2.0 (0/16)	11.6 ± 8.29 12.0 (4/24)	105.20 ± 13.10 110.00 (82/114)
MAS (*n* = 2)	0.00 ± 0.00 0.0 (0.0/0.0)	3.5 ± 0.71 3.5 (3/4)	109.50 ± 0.71 109.50 (109/110)
FD (*n* = 3)	1.00 ± 1.00 1.0 (0/2)	7.00 ± 2.65 8.0 (4/9)	109.67 ± 11.93 106.00 (100/123)
PHP (*n* = 6)	10.83 ± 10.26 12.0 (0/22)	7.83 ± 2.64 8.0 (4/11)	102.67 ± 14.36 101.50 (84/124)

Oral Health Impact Profile-14 (OHIP-14), Child Oral Health Impact Profile (COHIP), short form of Child Perceptions Questionnaire (SF-CPQ); OI, osteogenesis imperfecta OI; XHL, X-linked hypophosphatemic rickets; MAS, McCune–Albright syndrome; FD, fibrous dysplasia; PHP, pseudohypoparathyroidism; * higher COHIP values denote better OHRQoL.

## Data Availability

The dataset used and analyzed during the current study is available from the corresponding author on reasonable request.

## Supplementary Materials

The following supporting information can be downloaded at: https://www.mdpi.com/article/10.3390/ijerph21030306/s1, Table S1: Strobe checklist, Figure S1: Flow diagram of the cross-sectional study.
